# Numerical Optimization for the Geometric Configuration of Ceramics Perform in HCCI/ZTA_P_ Wear-Resistant Composites Based on Actual Particle Model

**DOI:** 10.1186/s11671-021-03514-1

**Published:** 2021-04-29

**Authors:** Ruiju Xu, Tianlong Lu, Jiankang Zhang, Yehua Jiang, Xiaoyu Chong, Jing Feng

**Affiliations:** 1 Faculty of Materials Science and Engineering, Kunming University of Science and Technology, Kunming, 650093 China; 2 Kunming Institute of Physics, Kunming, 650223 China; 3 Sino-Precious Metals Holding Co., Ltd., Kunming, 650106 China

**Keywords:** Wear-resistant composites, Finite element analysis, Equivalent grain model, Thermal stress, Compressive stress

## Abstract

In order to reduce the thermal stress in high chromium cast iron (HCCI) matrix composites reinforced by zirconia toughened alumina (ZTA) ceramic particles, finite element simulation is performed to optimize the geometric configuration of ceramics perform. The previous model simplifies the overall structure of the ceramic particle preform and adds boundary conditions to simulate the particles, which will cause uncontrollable error in the results. In this work, the equivalent grain models are used to describe the actual preform, making the simulation results closer to the actual experimental results. The solidification process of composite material is simulated, and the infiltration between molten iron and ceramic particles is realized. Thermal stress in solidification process and compression stress distribution are obtained. The results show that adding 10-mm round holes on the preform can improve the performance of the composite, which is helpful to prevent the cracks and increases the plasticity of the material.

## Introduction

With the continuous advancement of the industrialization process, traditional single wear-resistant materials have gradually become difficult to meet the performance requirements of wear-resistant parts in the fields of metallurgy, electric power, and building materials [1, 2]. Ceramic particles reinforced metal matrix composites, such as high chromium cast iron (HCCI) matrix composites reinforced by zirconia toughened alumina (ZTA) ceramic particles (referred as HCCI/ZTA_P_ composites hereinafter), are one of the most popular wear-resistant materials, which perfectly combines the high hardness of ZTA ceramic with the outstanding toughness of HCCI and makes full use of the complementary relationship between the two, giving the excellent wear resistance to metal matrix composites [3, 4].

HCCI/ZTA_P_ composites still have some cracking tendency, which may affect the appearance and stability of the production [5–7]. The cracking of composite materials is related to the plasticity and stress condition. Excellent plasticity and lower thermal stress can reduce the possibility of cracking of composite materials [8]. If the difference of the thermal expansion coefficient between ceramic particles and metal is too large, the thermal stress in composites will increase accordingly. When the thermal stress is high, cracks may be initiated inside the composite, especially at the interface between the ceramic particles and the metal. The continuous extension and propagation of cracks may eventually lead to the fracture of the composite material or even the entire layer peeling off [9, 10]. HCCI/ZTA_P_ composites materials also have the above problems. When the molten metal infiltrates into the aggregated particles, the temperature decreases, resulting in a poor combination ability of metal with ceramic particles. Therefore, in order to further improve the performance of HCCI/ZTA_P_ composites, it is important to study and reduce their cracking tendency [11, 12].

In HCCI/ZTA_P_ composites, the composite layer is designed as the working face and the rest matrix is metal, which makes the composite to have high wear resistance and plasticity at the same time [13]. The composite layer is prepared by the infiltration method in HCCI/ZTA_P_ composite. One of the remarkable characteristics of composite materials is their designability [14]. According to actual demand, the ceramic particles are prepared into a preform with a special structure and size, and then the preform is closely combined with the molten metal to prepare a ZTA_P_/HCCI composite [15].

In order to reduce the thermal stress, we choose the ceramic particles preform of hexagon. In the hexagonal preform, the maximum distance the molten metal penetrates the preform is the same regardless of the direction, so the uniformity of metal penetration is improved and the stress concentration in the preform is reduced [16, 17]. Although the hexagonal preform is used to reduce the tendency of the material to crack, the thermal stress in the material molding process still exists. Improving the structure of the ceramic particle preform can effectively reduce stress concentration.

In the optimization of composite preform structure, the finite element method can reduce repeated experiments. In previous studies, due to the complexity of ceramic particle drawing and calculation, the ceramic particle preform is usually simplified as a whole. Thorough research found that the simplified model has some defects and cannot be used in a wider range. The establishment of equivalent particle model can further combine the model with the actual situation and reduce the error caused by the model [18]. The finite element analysis software COMSOL Multiphysics method is used to model the stress fields in the solidification process and compression process of the HCCI/ZTA_P_ composite material [19]. COMSOL Multiphysics is a large-scale advanced numerical simulation software [20, 21].

In the paper, we use finite element software to simulate the stress of composite materials under different conditions. The version of COMSOL Multiphysics used in this paper is 4.5a. This study systematically analyzes the influence of the geometric model in the finite element software on the calculation results, which benefits the design and development of porous perform. The simulation and experiment are compared with each other, and the model is continuously optimized.

## Methods

One of the major issues in composites production is stress concentration, and it directly affects the wear resistance and plasticity of composite materials. Improving the structure of the ceramic particle preform can effectively reduce stress concentration. The purpose of this study is to investigate the influence of the preform structure on the stress distribution and improve the performance of composite materials.

### The Establishment and Optimization of Geometric Model

In the process of solidification, the temperature of mold and liquid metal is different and liquid metal solidifies rapidly, so the heat transfer between each position in the casting process is unsteady, and the heat transfer equation can be written as [22]:

1 $$\rho C_{{\text{P}}} \frac{\partial T}{{\partial x}} = \frac{\partial }{\partial x}\left( { \lambda \frac{\partial T}{{\partial x}}} \right) + \frac{\partial }{\partial y}\left( { \lambda \frac{\partial T}{{\partial y}}} \right) + \frac{\partial }{\partial z}\left( { \lambda \frac{\partial T}{{\partial z}}} \right) + \rho Q$$

where $\rho$ is density; $C_{{\text{P}}}$ is heat capacities; $\lambda$ is thermal conductivity; *T* is transient temperature; *Q* is heat; the coordinates *x*, *y*, and *z* are called the relative coordinates of subsystem.

Since the temperature of each point is different in the solidification process, there is a variable internal stress in the casting. If the casting can be regarded as linear elasticity body, when the internal stress is less than the yield limit, with the process of elastic deformation, we can use Hooke's law equation to describe it.

2 $$\left\{ {\begin{array}{*{20}l} {\varepsilon_{xx} = \frac{1}{E}\left[ {\sigma_{xx} - v\left( {\sigma_{yy} + \sigma_{zz} } \right)} \right]} \hfill \\ {\varepsilon_{yy} = \frac{1}{E}\left[ {\sigma_{yy} - v\left( {\sigma_{xx} + \sigma_{zz} } \right)} \right]} \hfill \\ {\varepsilon_{zz} = \frac{1}{E}\left[ {\sigma_{zz} - v\left( {\sigma_{xx} + \sigma_{yy} } \right)} \right] \to \varepsilon_{ij} = \frac{1 + v}{E}\sigma_{ij} - \frac{v}{E}\delta_{ij} \sigma } \hfill \\ {\varepsilon_{xy} = \frac{1}{2G}\sigma_{x} } \hfill \\ {\varepsilon_{yz} = \frac{1}{2G}\sigma_{yz} } \hfill \\ {\varepsilon_{zx} = \frac{1}{2G}\sigma_{zx} } \hfill \\ \end{array} } \right.$$

where *E* is Young's modulus; $\sigma = \sigma_{{ii + \sigma_{11} }} + \sigma_{22 + } \sigma_{33}$; $v$ is Poisson's ratio; unit tensor $\varepsilon_{ij} = \frac{1}{2}\gamma_{ij}$; shear modulus $G = \frac{E}{{2\left( {1 + v} \right)}}$.

Then the internal stress is larger than the yield limit, and the casting has more deformation. Total strain is composed of elastic strain and plastic strain, $\sigma_{ij} = \sigma_{ij}^{e} + \sigma_{ij}^{p}$. This equation can be treated as elastic plastic linear hardening model. Elastic deformation and plastic deformation are linear, and the constitutive equation can be written as [23]:

3 $$\sigma = \left\{ {\begin{array}{*{20}l} {E\varepsilon } \hfill & {\varepsilon \le \varepsilon_{{\text{s}}} } \hfill \\ {\sigma_{{\text{s}}} + E_{1} \left( {\varepsilon - \varepsilon } \right)} \hfill & {\varepsilon > \varepsilon_{{\text{s}}} } \hfill \\ \end{array} } \right.$$

where $\sigma$ is strain; *E* is Young's modulus; $\varepsilon$ is stress; $\varepsilon_{{\text{s}}}$ is yield strength.

The service life of high chromium cast iron workpieces is shorter due to poor wear resistance. Composite materials have many advantages over single HCCI. In the manufacturing process of HCCI/ZTA_P_ composites, the ZTA ceramic particles are prepared in advance into a porous preform. The preform makes the ZTA ceramic particles and the HCCI more tightly combined, and the ceramic particles are not easy to fall off when subjected to impact. In order to further improve the performance of the HCCI/ZTA_P_ composites, it is necessary to optimize the structure of the preforms.

During the casting process, the molten metal can fill the circular holes and increase the proportion of metal. In the application process of HCCI/ZTA_P_ composites, it is found that the particle aggregation position is more prone to crack, so the circular hole is added in this position.

**Fig.1:**
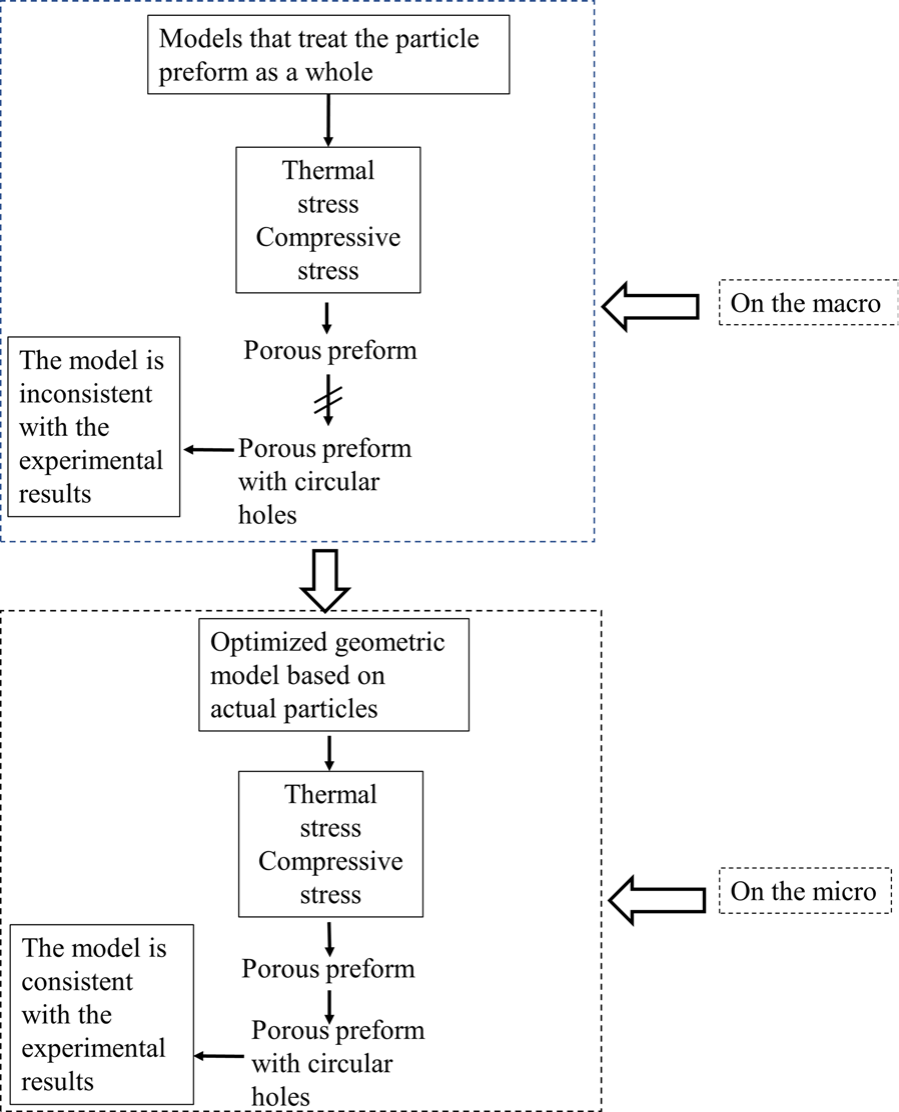
The flowchart of geometric model optimization for HCCI/ZTA_P_ materials

The shape of ZTA ceramic particles is arbitrary polygon, and there are many particles in preform. If all the particles are drawn, the workload is large. In previous simulations of ceramic metal matrix composites, most of the preforms of ceramic particles were considered as a whole. As shown in Fig. 1, the paper establishes models on the macroscale and microscale, respectively. The establishment of a reasonable model requires repeated mutual verification with experiments. In the optimization model, the calculated results are in good agreement with the experimental results. The simulation results included thermal stress, temperature field, phase transition field during solidification and stress strain of the casting under load. Make reasonable and effective adjustments to the established finite element model so that the optimized finite element model can be used in a wider range.

**Fig.2:**
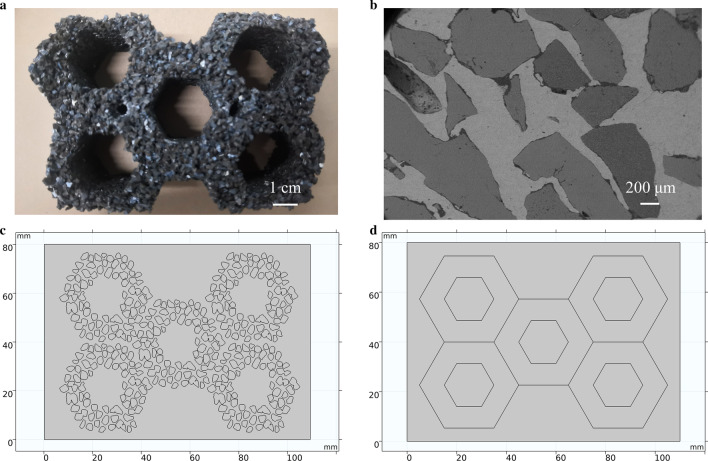
ZTA ceramic particles and geometric model of HCCI/ZTA_P_ composites. **a** Porous perform, **b** the composite zone of HCCI/ZTA_P_ composites, **c** optimized geometric model based on actual particles, **d** initial geometric model

In the establishment of the geometric model using the finite element software, in order to reduce the calculation time and the modeling workload, the simplified geometric model is often used. As shown in Fig. 2, there are gaps between the particles because the three-dimensional hexagonal porous preform is simplified to a two-dimensional model, and only one layer of particles is selected to project the two-dimensional model. In this way, the stacking of particles in the three-dimensional space can effectively avoid the influence on the two-dimensional geometric model and reasonably simplify the model and improve the calculation efficiency.

In this paper, ZTA ceramic particles are selected as the reinforcing phase of the composite. ZTA ceramic particles are a multiphase structural ceramic prepared by adding zirconia to alumina and then sintering, where in the zirconia mass fraction is 18% and the alumina mass fraction is 82%. High chromium cast iron is the matrix of composite material, which contains more than 12% chromium, and is an excellent wear resistant material.

The material parameters have great influence on the result of finite element calculation. The material parameters required for the simulation calculation in this paper are obtained by experiments and literature. The material parameters of ZTA ceramic particles and HCCI are shown in Table 1.

**Table 1:** Material parameters

Temperature/℃	*E*/GPa		(μ)		*α* (10^–5^/℃)	
	ZTA	HCCI	ZTA	HCCI	ZTA	HCCI
25	3.00	205	0.245	0.291	0	0
700	2.95	140	0.248	0.316	0.9	1.386
1300	2.82	0	0.252	0.500	0.91	2.370
1570	2.75	0	0.258	0.500	0.92	2.675

*E*—plastic modulus, *μ*—Poisson's ratio, *α*—coefficient of thermal expansion

### Experiments

For testing the plasticity and wear resistance of the HCCI/ZTA_P_ composite material, a systematic test was carried out on the composite to further determine the influence of the preform structure. SHT4305 universal testing machine was used to measure the compression properties of HCCI/ZTA_P_ composites. The size of the compression test sample is 10 × 10 × 25 mm, the applied load is 30 tons, and the compression speed is 0.5 mm/min.

The wear resistance test of HCCI/ZTA_P_ composite is necessary. Excellent abrasion resistance is the most important criterion for testing the performance of wear-resistant composite materials. HCCI/ZTA_P_ composites are mainly used in the mineral processing, cement manufacturing, and paper manufacturing industries, and most of the working conditions are three-body abrasive wear. In order to simulate the service performance of HCCI/ZTA_P_ composites under actual working conditions as much as possible, tested the HCCI/ZTA_P_ composites using MMH-5 three-body abrasive wear tester. The track material of the tester is M2 tool steel, the hardness 820–860 Hv, outer diameter 380 mm, width 20 mm. The type and size of abrasives are selected according to different working conditions. In this paper, quartz sand is used for abrasives, the hardness is 1000–1200 Hv, the test load is 40 N, and the sample rotating speed is 30 r/min.

A nanoindenter is used to perform a 100-point Young's modulus test in the selected 100 × 100 μm^2^ microregion. The model of the nanoindenter is iMicro.

The wear resistance of materials can be measured with mass reduction, volume reduction, and so on. The volume loss measured by a measuring cylinder with small changes can easily cause errors in human readings. Therefore, under the same wear conditions, the mass loss Δm is used to evaluate the wear performance of the material. The formula for calculating material loss is as follows:

4 $$\Delta m = m_{1} - m_{2}$$

where *m*_1_ and *m*_2_, respectively, represent the mass of samples before and after wear.

## Results and Discussion

### Simulation Based on Simplified Entire Model

**Fig.3:**
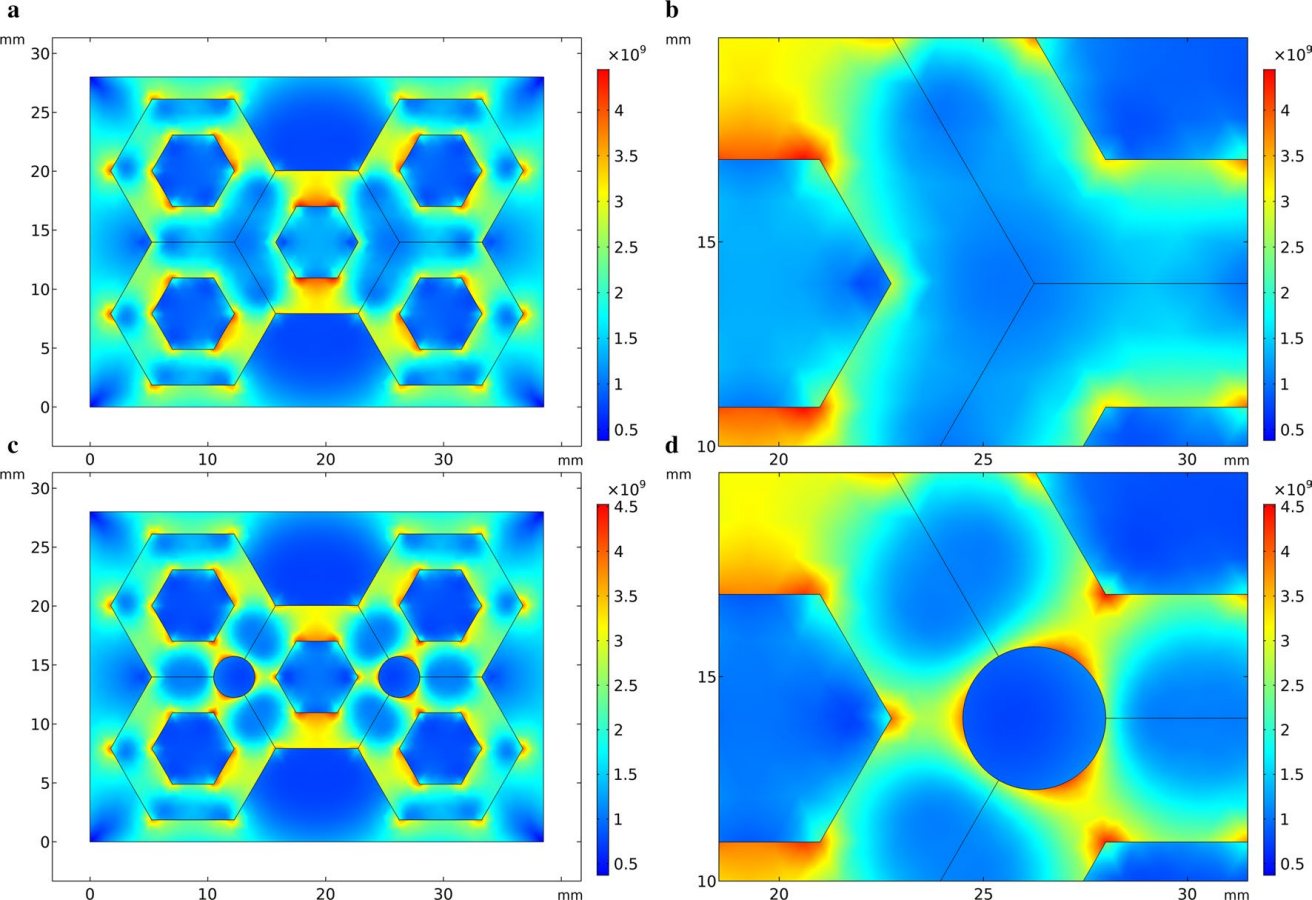
Thermal stress distribution during solidification in the simplified model. **a** Initial perform, **b** partial enlarged view of the initial perform, **b** preform with circular holes added, **d** partial enlarged view of the perform with circular holes added

**Fig. 4:**
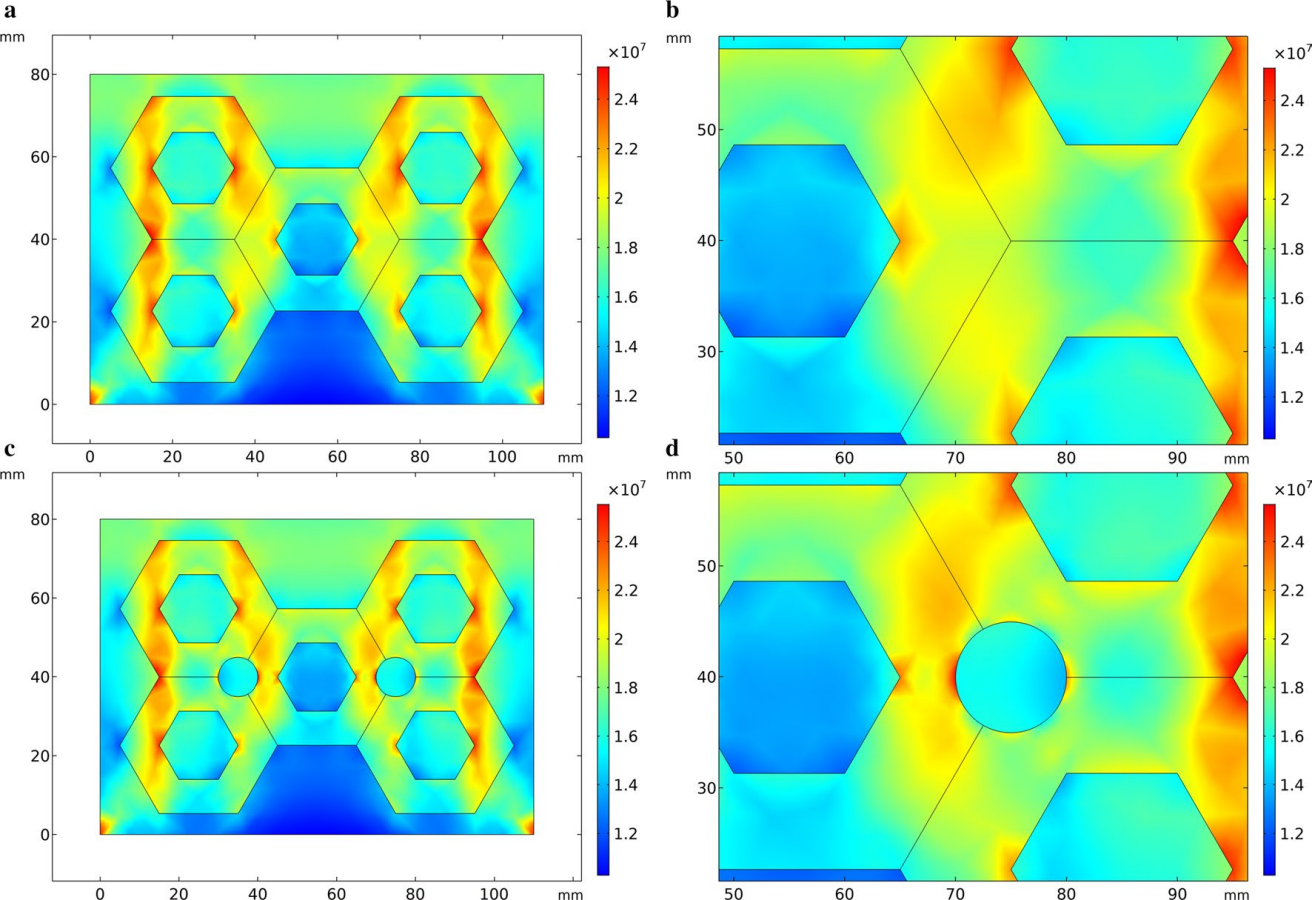
Compressive stress in the simplified model. **a** Initial perform, **b** partial enlarged view of the initial perform, **b** preform with circular holes added, **d** partial enlarged view of the perform with circular holes added

In the simulation of thermal stress in the solidification process of HCCI/ZTA_P_ composites in this study, the thermal stress distribution at 10 s is selected for all the simulation results, because the thermal stress changes significantly before and after 10 s. Compared with the scale at the right of Fig. 3, red color indicates higher stress and blue color indicates lower stress. In Fig. 3a, stress concentration appeared on the edge of the preform, especially in the middle position, the upper side and the lower side of the preform appeared red. Comparing to the right scale of Fig. 3a, it shows that the stress is enormous here. Blue color appears at the place where the particles gather, that is, the intersection of the hexagonal hole walls, indicating that the stress is small here. In the geometric model in Fig. 3c, circular holes are added to the particle aggregates of the preform. The stress distribution in Fig. 3c is similar to Fig. 3a, except that there is a more obvious stress concentration around the circular holes. The stress distribution around the circular hole of the perform in Fig. 4 is similar to that in Fig. 3

**Fig. 5:**
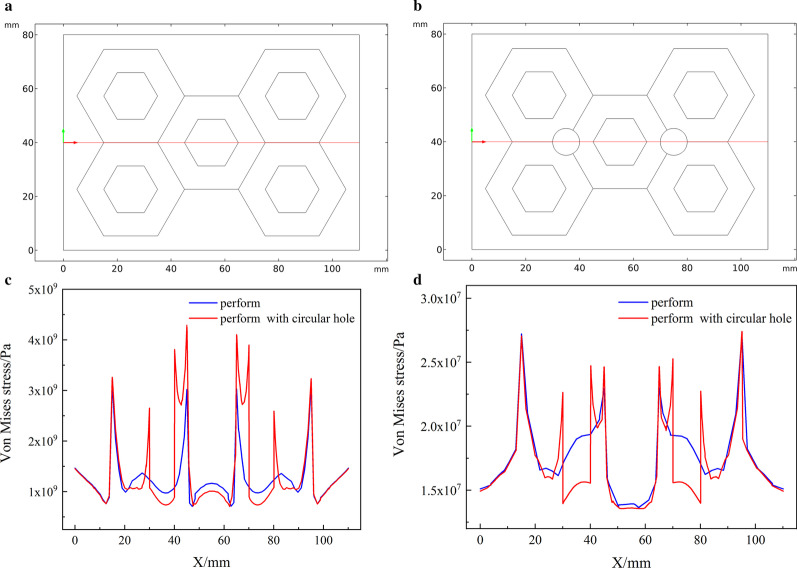
The position of the 2D transversal of the simplified model and stress transversal comparison. **a** Initial perform, **b** partial enlarged view of the initial perform, **c** preform with circular holes added, **d** partial enlarged view of the perform with circular holes added

The final calculation results need intuitive, so the results were post-processed and a stress transversal comparison chart was drawn. First draw a 2D transversal in the geometric model, because the main observation part is around the circular hole, that is, where the particles gather, so the 2D transversal passes through the circular hole. The ordinate of the stress graph is the stress value on the section line, and the abscissa is the *x*-axis coordinate of the model, as shown in Fig. 5. In the simplified model, the circular hole coordinates are (12, 14), (27, 14). Figure 5c is a line graph of solidification stress. In Fig. 5c, the preform with circular holes has a significant increase in the stress at the abscissas 12 and 27, compared with the preform without circular holes. Figure 5d is a comparison chart of compressive stress. The general trend of the curve is the same as that of Fig. 5c, and the location stress of the circular hole will increase significantly.

### Simulation Based on Equivalent Grain Model

**Fig. 6:**
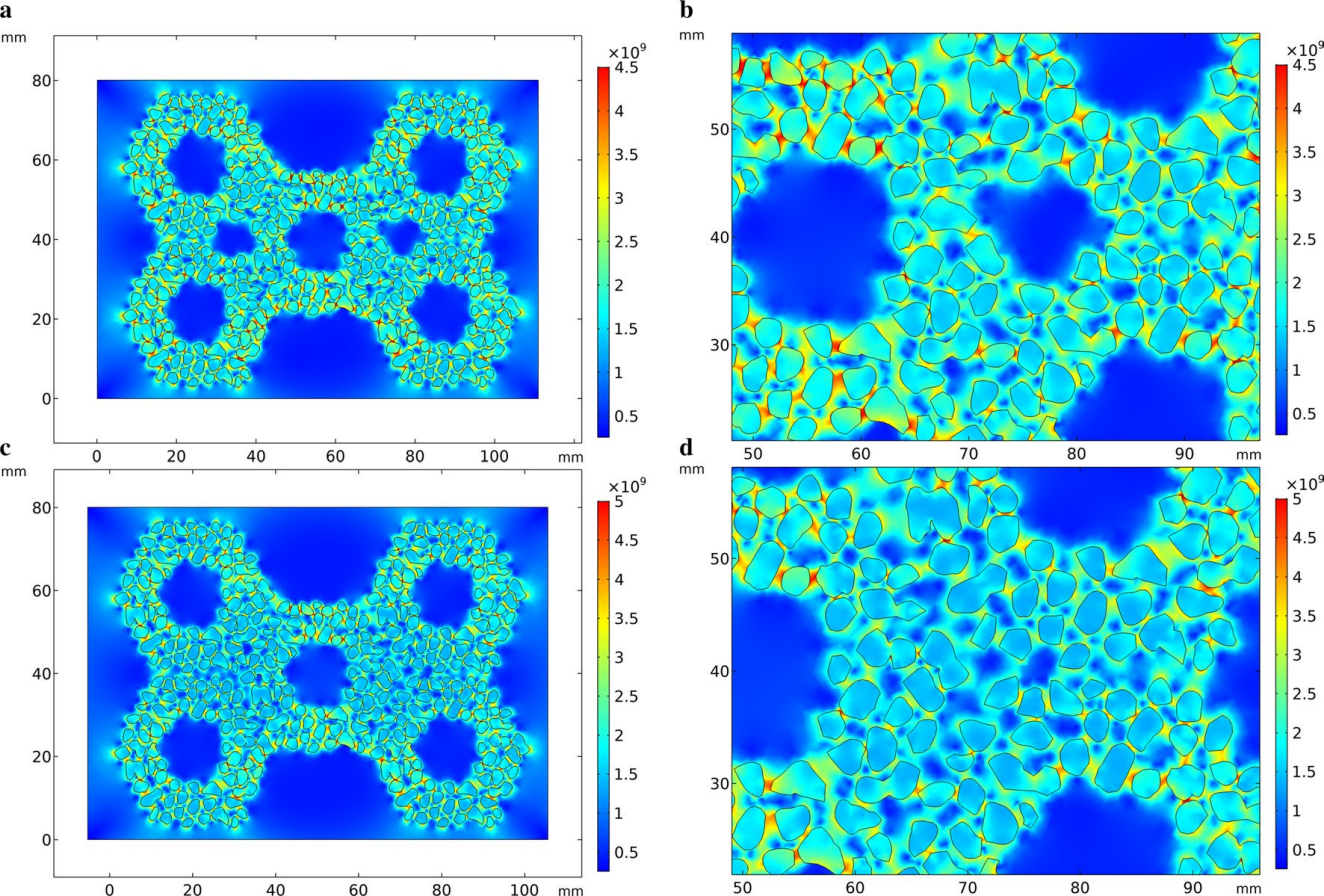
Thermal stress distribution during solidification in the optimized model. **a** Initial perform, **b** partial enlarged view of the initial perform, **c** preform with circular holes added, **d** partial enlarged view of the perform with circular holes added

Figure 6 shows the thermal stress distribution of HCCI/ZTA_P_ composites model based on actual particles, which is similar to Fig. 3. However, in Fig. 6, the ZTA ceramic particles are no longer simplified as a whole preform, but established as individual particles, and it can be observed that most of the particles around appear red color. The shape of ZTA ceramic particles is not uniform, and the stress is higher than the surrounding value, especially at the sharp point of the particles. The thermal stress distributions in Figs. 3b and 6b are obviously different. The circular hole of the preform in Fig. 6b appears blue color, indicating that the stress is small here. The calculation results of stress concentration around the circular hole are opposite. The degree of simplification and drawing methods of geometric models lead to different calculation results.

**Fig. 7:**
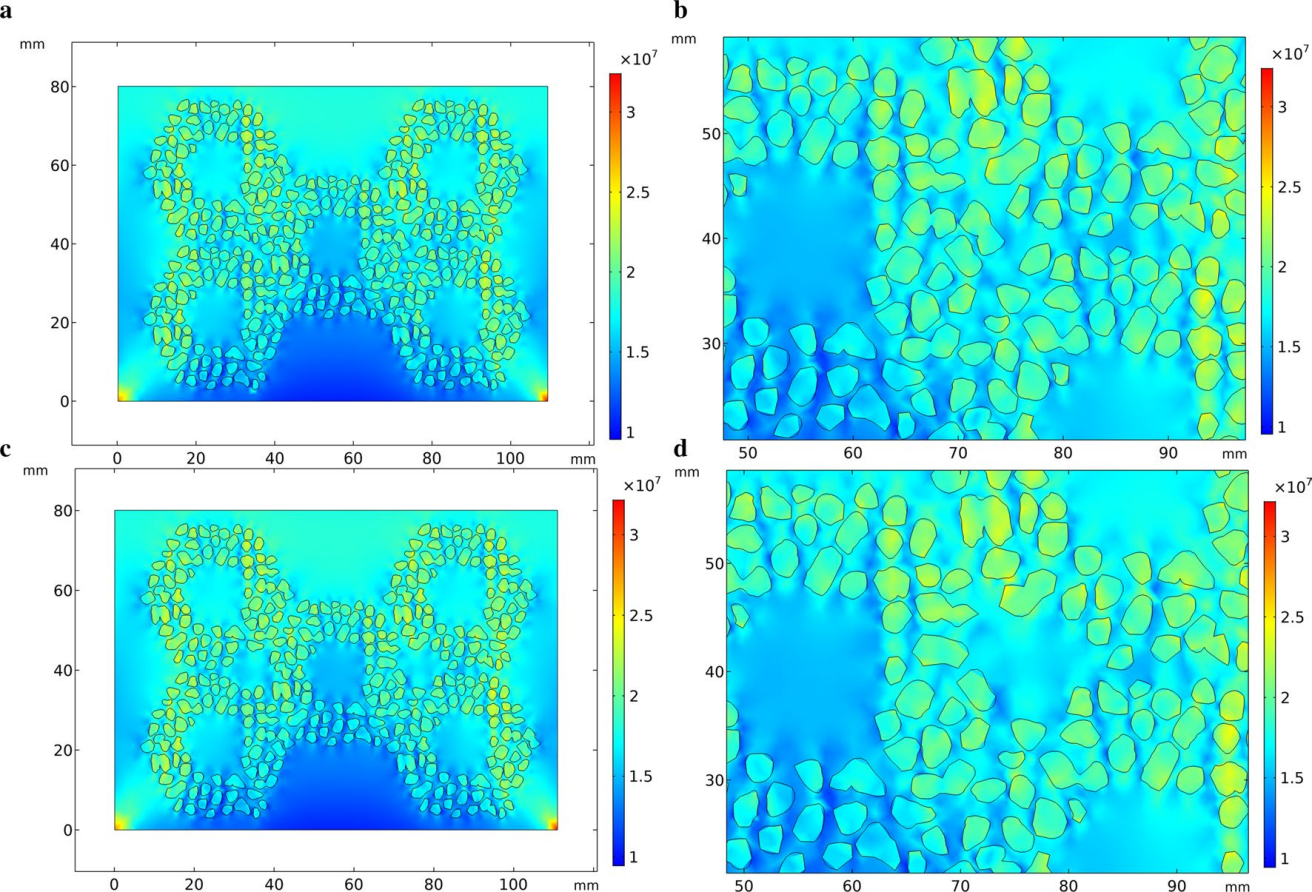
Compressive stress in the optimized model. **a** Initial perform, **b** partial enlarged view of the optimized perform, **c** preform with circular holes added, **d** partial enlarged view of the perform with circular holes added

The geometric model used to simulate the compressive stress in Fig. 7 is the similar as Fig. 6. In Fig. 7, comparing with the scale on the right, the stress is concentrated on the upper part of the model and the preform, especially the edges on both sides of the ceramic preform, which are yellow-green. In the particle aggregation part of the preform, this zone is shown in green color in Fig. 7a, indicating that the stress is small here. In Fig. 7b, circular holes are added at the aggregates of the preform. The color of the circular holes zone is green and yellow, indicating that there is no obvious stress concentration.

**Fig.8:**
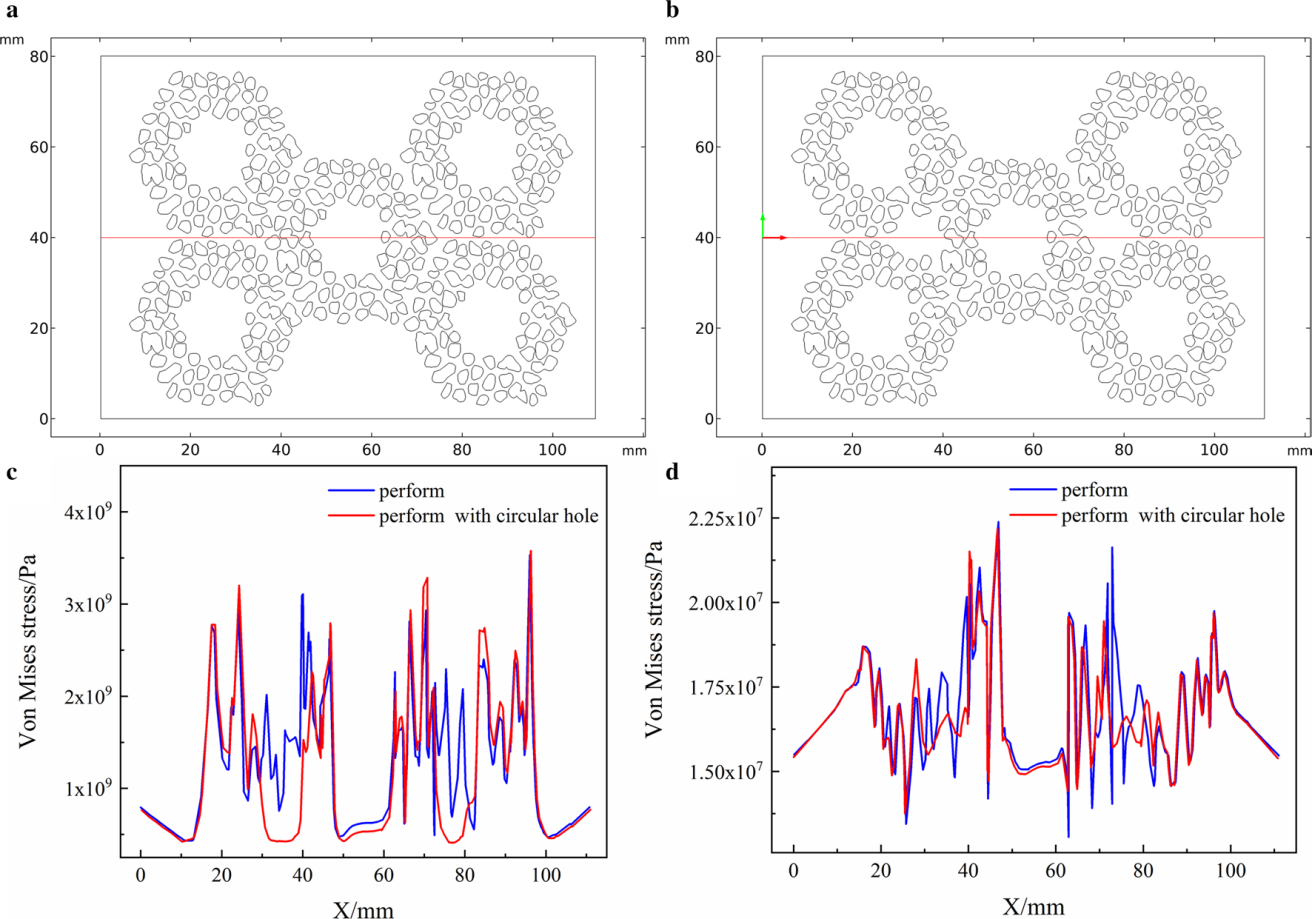
The position of the 2D transversal of the optimized model and stress transversal comparison. **a** Initial perform, **b** preform with circular holes added, **c** solidification stress, **d** compression stress

In the model based on actual particles, the circular hole coordinates are (12, 14) (27, 14). Figure 8 is a comparison diagram of solidification stress, comparing the effect of the presence or absence of circular holes on stress. The stress of the preform with circular holes showed a significant reduction at the abscissas 12 and 27, and its position basically coincided with the position of the circular holes. The stress of the remaining coordinates of the preform with circular holes has a small increase. In Fig. 8a, b, the two curves are basically coincident, except in the preform with circular holes; the stress near the circular hole coordinates drops significantly.

### Experimental Validations

**Fig. 9:**
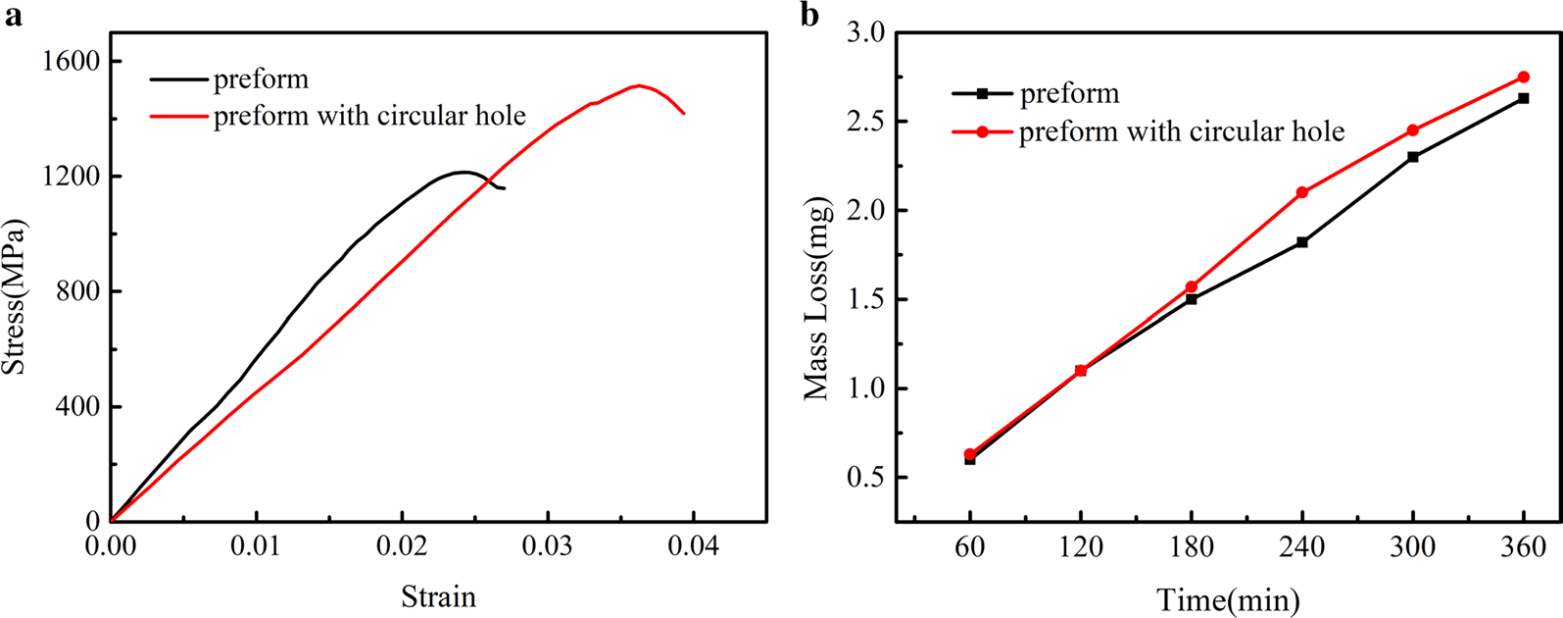
Compression stress–strain curve of HCCI/ZTA_P_ composite material and mass loss of three-body abrasive at the junction of composite honeycomb walls

It can be seen from Fig. 9, in the wear test, the mass loss of the composites using the optimized preform and the composites with the original preform is not much different, indicating that the wear resistance is not significantly sacrificed and can also improve the overall plasticity of the HCCI/ZTA_P_ composites.

The porous preform was optimized by adding small size cylindrical holes at the particle aggregation zones, which changed the volume fraction of ceramic particles in the HCCI/ZTA_P_ composites. The content of ZTA ceramic particles in the composite material is an important factor affecting its mechanical properties. As shown in Fig. 9, the compression strength and compression deformation of sample which has preforms with circular holes increase significantly compared with that of sample with the initial preform, indicating that small circular holes at the aggregation zones of ZTA ceramic particles are conducive to improving the strength and plasticity of the HCCI/ZTA_P_ composites. The addition of small cylindrical holes at the agglomeration zones of porous preform will increase the content of the metal matrix, thus increasing the compressive strain of the HCCI/ZTA_P_ composites under compressive stress and also affecting its compressive strength. When the stress reaches the peak value, it can be considered that the damage has already occurred in the specimen. With the continuous increase in strain, the internal damage of the material is also accumulating, the strain resistance gradually decreases, and finally the shear failure occurs.

**Fig. 10:**
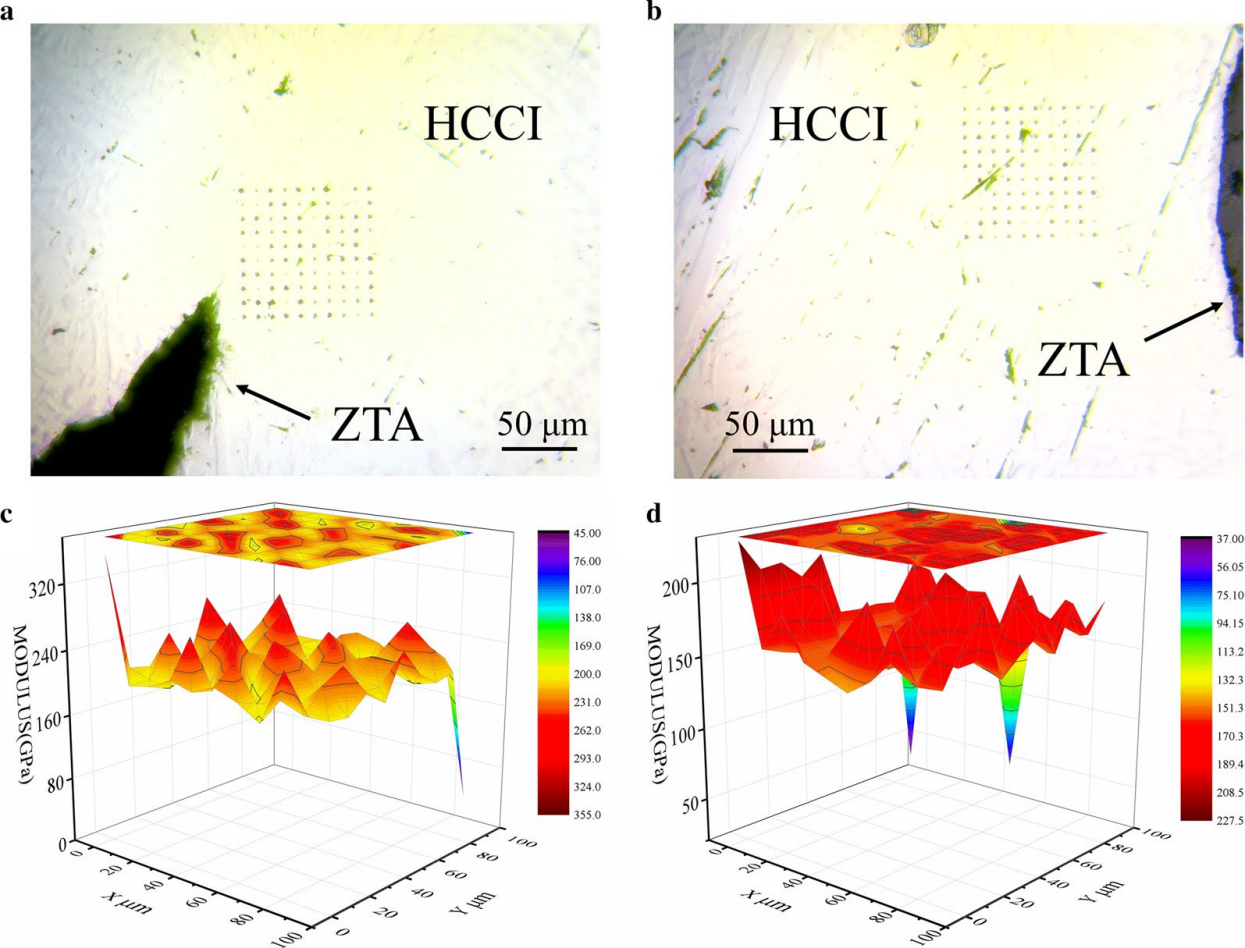
Indentation distribution of composite materials and the distribution of Young's modulus around the particles of composite materials. **a** The shape of the particles is sharp, **b** the shape of the particles is rounded, **c** the shape of the particles is sharp, **d** the shape of the particles is rounded

The microanalyses of hardness of all samples are shown in Fig. 10. As shown in Fig. 10a, b, the test area is selected around the rounded particles and sharp-cornered particles, respectively, so as to better correspond to simulation results. Figure 10c, d is compared with the enlarged partial view of stress. In the simulation, stress concentration tends to occur around sharp corners of the particles. In the test results, the modulus near the sharp corner particles is greater than the metal matrix near the round corners, which further verifies the rationality of the model based on the actual particle.

A circular hole is added to the particle aggregation zones of the preforms, which has three functions. The first function is to reduce the volume fraction of ceramic particles in the HCCI/ZTA_P_ composite material and reduce the residual stress; the second function is to reduce the agglomeration of the ceramic particles of the preform; the third function is to increase the volume fraction of the metal matrix with better plasticity to hinder crack propagation. The plasticity of HCCI/ZTA_P_ composites decreases with the increase in residual stress. The volume fraction of ceramic particles decreases, and the residual stress becomes relatively small. The addition of circular holes in the ZTA ceramic particle preform can reduce cracks because the crack instability tends to extend along a straight line. When cracks are generated in the composite zone of HCCI/ZTA_P_ composite material, the cracks are easily extended along the hexagonal hole wall in the preform to generate crack propagation. The circular hole increases the content of HCCI matrix at the junction of hexagonal hole wall, hinders the crack propagation, and thus plays a role in toughening the structure.

## Conclusion

The large difference in thermal expansion coefficient between HCCI and ZTA_P_ causes cracks in the composite material. The solidification and compression process of HCCI/ZTA_P_ composite materials are simulated by using the finite element software, and the numerical values and distributions of stresses analyzed based on simplified entire model and equivalent grain model for preform. According to the calculation results, the preform structure is optimized. By analyzing the simulation and experimental results, it can be concluded that the addition of circular holes to the hexagonal porous preform will lead to a decrease in thermal stress and compressive stress during solidification. After the optimization and adjustment of the model, the simulation results tend to be consistent with the experimental results. The methods in this paper may provide the important reference for the simulation and optimization of processing parameters in casting systems of the various metal matrix composite.

## Data Availability

The datasets supporting the conclusions of this article are included within the article.

## Abbreviations

HCCI: High chromium cast iron
ZTA: Zirconia toughened alumina
HCCI/ZTA_P_: High chromium cast iron matrix composites reinforced by zirconia toughened alumina ceramic particles composites
